# Marketing analytics in banking 4.0: A two-stage explainable AI framework for high-accuracy and well-calibrated predictions

**DOI:** 10.1371/journal.pone.0348767

**Published:** 2026-05-15

**Authors:** Fahim Nasir, Abdulghani Ali Ahmed, Iryna Yevseyeva, Mehmet Sabir Kiraz

**Affiliations:** Digital Futures Institute, School of Computer Science and Informatics, De Montfort University, Leicester, United Kindom; Macao Polytechnic University, MACAO

## Abstract

Forecasting customer conversion in bank marketing is challenged by imbalanced class distributions, where scarce minority responses lead to underfitting of true patterns or overfitting of limited instances. Sampling techniques are commonly applied to address this class imbalance problem; however, synthetic data generation may introduce distributional distortion, inflate apparent performance while degrade calibration, fairness, and explainability. Finding and selecting suitable model-sampling combinations that transparently balance performance against distortion remains a challenging task. To address this, we propose a two-stage quadrilateral evaluation framework that assesses models with sampling techniques across discrimination, calibration, computational cost, and explainability to select the suitable. In the first stage, we test ensemble learning and deep learning models on datasets with imbalanced class distributions using k-fold cross-validation and hyper-parameter tuning. In the second stage same models were re-evaluated under five sampling strategies, including Borderline2Smote, to quantify the trade-offs introduced by synthetic sampling. Results show that the combination of XGBoost with Borderline2SMOTE sampling demonstrates improved adaptability with reduced synthetic distortion. Shapley Additive exPlanations (SHAP) further support stable interpretability by identifying key drivers of customer responses for predictive inference and marketing strategies. This framework enables context-aware, transparent, and reproducible model selection strategy for responsible predictive analytics in Banking 4.0. This study also provides a foundation for further research into fairness-aware and explainable predictive analytics to support data-driven decision-making.

## 1. Introduction

Banking 4.0 represents the next evolution from the digital accessibility of Banking 3.0, integrating advanced artificial intelligence (AI) for data-driven decisions. This shift enables intelligent automation and highly personalised services, but it also demands a foundation of responsible AI, built on ethical principles and regulatory compliance. The potential is vast, with projections suggesting AI could generate over $28 trillion in global financial revenue by 2030, a value already demonstrated by its strategic use in major banks like Barclays and JP Morgan Chase [1].

However, much of the current research focuses heavily on predictive accuracy. Our study addresses a critical gap by examining how sampling techniques, computational efficiency, and model calibration must work together to ensure fairness, transparency, and regulatory alignment. We argue that in the context of Banking 4.0, these factors are not secondary concerns but are just as vital as accuracy itself for building trustworthy and effective AI systems [2–5]. This study frames Banking 4.0 as the operationalisation of Responsible AI in marketing, unifying predictive performance, fairness, explainability, and compliance. A central bottleneck is the use of imbalanced customer conversion data, where rare positive responses can distort predictions, waste resources, and complicate compliance. This highlights the lack of a unified evaluation framework to quantify the balance among performance, interpretability, and practical efficiency for banking’s data challenges. Therefore, this research is motivated to develop a systematic approach for optimising and explaining AI predictions on imbalanced data, and quantifying the impact of sampling techniques. It also evaluates adaptability due to technical overheads with ethical governance [6–8].

The banking industry possesses a robust technological infrastructure that manages vast structured data comprising transactions and customer interactions. This data foundation enables the use of AI for predictive analytics in key areas like risk management, fraud detection, and personalised marketing [9–11]. Marketing is a complex and multi-faceted process that encompasses a wide range of challenges, from understanding customer behaviour to optimising campaign strategies. Banks deploy machine learning (ML) for customer segmentation, churn prediction, cross-selling, personalised services, and real-time campaign optimisation. Predictive modelling in ML enhances return on investment (ROI) and engagement. Integrating explainable AI (XAI) framework in predictive modelling ensures regulatory compliance and ethical deployment in bank marketing campaigns. It provides transparent reasoning behind decisions for segmentation, pricing, and churn reduction etc. As exemplified by cross-sector applications like Amazon’s recommendation engines. This synergy of data-driven insights and explainability fosters customer trust, competitive agility, and sustainable growth in marketing [12,13]. Some marketing problems outlined in Table 1 below, along with multiple deployed case-studies across various industries, highlight how AI-driven predictive analytics can address these challenges. These real-world case-studies demonstrate how AI is being leveraged to improve customer segmentation, optimise marketing campaigns, and drive revenue growth across diverse sectors.

**Table 1:** Marketing challenges and AI-driven predictive solution with deployed case studies.

Marketing Problem	Challenges in Predictive Analytics	AI-Powered solutions with case studies
Marketing campaigns	Aligning AI predictions with evolving consumer demographic backgrounds.	*Uber* uses AI algorithms to segment customers and offer personalised promotions, discount on next ride [13].
Customer behaviour and preferences	Predicting customer preferences based on consumer buying behaviour.	*Salesforce’s Einstein* AI analyses customer behaviour and preferences to automatically direct customers to suitable agents [13].
Integrating business processes	Unifying cross-channel marketing efforts for a seamless strategy.	*Coca-Cola* integrates AI-based sentiment analysis with real time promotions [13].
Analysing market trends	Processing large-scale data to make real-time marketing decisions.	*Amazon* uses AI to analyses user interaction and predicts product demand [12].
Extracting Actionable insights	Ensuring AI-generated insights align with business objectives.	*Netflix’s* AI recommendation engine boosts engagement by predicting user preferences [12].
Maximising revenue with customer retention	Predicting customer lifetime value and improving loyalty programs	*AT& T* and *Verizon* use AI to forecast customer lifetime value to proactively anticipate churn risks [13].

The transformative potential of AI in marketing, as demonstrated in Table 1, extends prominently to the banking sector, where predictive analytics drives customer segmentation, lead scoring, and real-time campaign optimisation, enabling precision-targeted strategies [12,13]. AI-powered tools including dynamic pricing, automated customer support, and hyper-personalised engagement systems, leverage structured tabular datasets, compromising customer demographics, transaction histories, credit scores, and financial interactions to predict needs, identify upsell opportunities, and assess risks. However, such datasets often exhibit severe class imbalance, particularly in marketing campaigns where positive responses are rare, skewing predictions towards majority due to class bias. Mitigating this necessitates advanced sampling techniques to recalibrate data distributions, ensuring classifiers yield actionable insights for ethical and accurate decision-making in banking [14].

Classification is a fundamental machine learning task for assigning categories to data. Multiple recently published related work shows that Gradient Boosted Decision Trees (GBDTs) often outperform them on structured, imbalanced data. GBDTs provide strong accuracy with greater efficiency and simplicity. While Deep Learning and LLMs excel with unstructured data [5,15,16]. Ensemble methods enhance this by combining multiple models to improve accuracy and reduce overfitting. For example, Random Forest (RF) uses ‘bagging’ to decrease model variance, while Extreme Gradient Boosting (XGBoost) uses ‘boosting’ to sequentially correct errors and reduce bias. These ensemble models, including others like Adaptive Boosting (AdaBoost), Categorical Boosting (CatBoost), and Light Gradient Boosting Machine (LightGBM), are particularly effective for tabular data. They are robust, capable of handling imbalanced datasets, and generalise well to unseen data [10]. In contrast, Deep Learning (DL) uses multi-layered neural networks to automatically learn complex patterns from large, unstructured datasets like images and text. While models such as Convolutional Neural Networks (CNNs), and Multilayer Perceptron’s (MLP) excel in these domains. Their application to structured, tabular data is challenging. For instance, TabNet is a DL model designed for tabular data that uses an attention mechanism to select relevant features. However, advanced DL models, including TabNet and Large Language Models (LLMs), are prone to overfitting, especially on smaller datasets. They also require significant GPU resources and vast amounts of data to perform effectively. These constraints of computational cost, data availability, and overfitting make them less suitable for the typical, resource-constrained environments of banking data analytics [17,18].

Despite progress in predictive analytics, important gaps remain in the development of trustworthy AI for imbalanced tabular marketing data. Prior studies tend to focus mainly on discrimination metrics such as the area under the receiver operating characteristic curve (AUC-ROC) and the F1-score, while placing less emphasis on calibration and explainability, which are essential for ethical AI under the Banking 4.0 framework. Our proposed quadrilateral evaluation framework addresses these gaps by bringing together discrimination, calibration, computational efficiency, and explainability in a unified assessment suited to high-stakes environments. Also, by finding a suitable model-sampling combination that transparently balances performance against distortion from synthetic artefacts. This approach ensures that models are not only accurate but also reliable, practical, and transparent for audit and regulatory purposes. Sampling techniques such as the Synthetic Minority Oversampling Technique (SMOTE) and related variants are widely used to address class imbalance, but their influence on interpretability, computational behaviour, and bias inflation remains under-explored in the banking sector. DL classifiers, although powerful in theory, are rarely benchmarked against ensemble learning models with a focus on explainability in resource-limited environments. These limitations hinder progress towards practical, ethical, and auditable AI systems in banking, and they leave practitioners without clear guidance for real-world deployment [19–22].

This study aims to address the key gaps in imbalanced banking analytics by developing a systematic framework that aligns predictive performance, computational efficiency, and explainability with the requirements of Banking 4.0. Accordingly, this study makes the following contributions:

1) We introduce a reproducible two-stage framework that offers a quadrilateral evaluation of discrimination, calibration, computational behaviour, and explainability on both imbalanced and sampled datasets for marketing campaign predictions. It enables clear quantification of bias and model adaptability.
2) We present a simulation-based comparison that shows how EL can achieve performance and calibration comparable to DL models for marketing datasets, while maintaining superior computational efficiency, helping to resolve the performance-efficiency constraints seen in banking applications.
3) We examine the trade-offs and distributional shifts introduced by synthetic sampling, showing that focusing the generation on borderline minority instances (B2S) preserves local class structure more effectively than global oversampling. Examining how this affects feature importance patterns and the stability of SHAP-based explanations in predictive marketing analytics.

**Fig 1:**
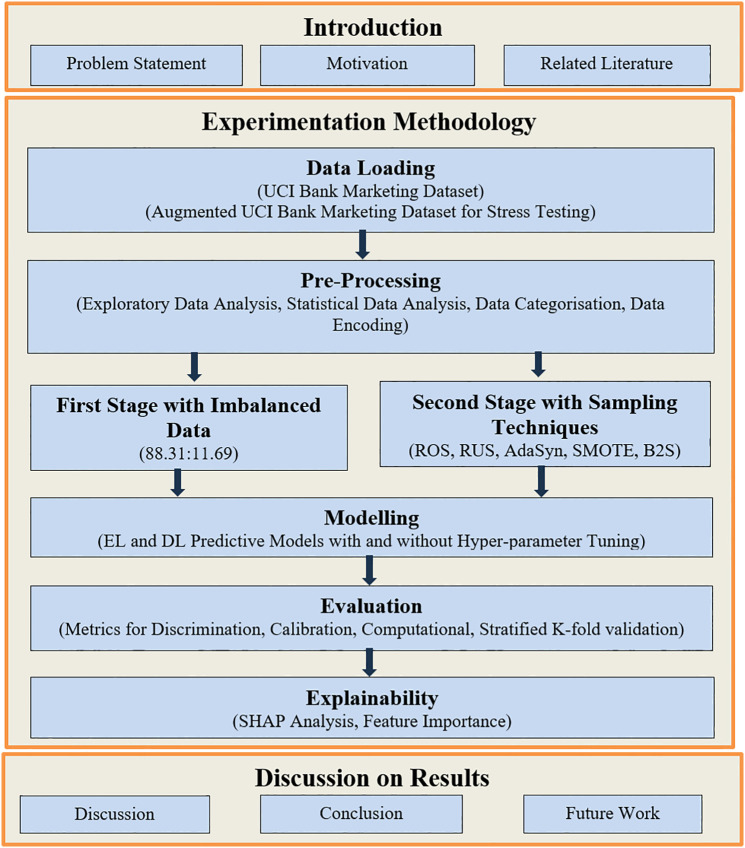
Overall workflow of this study.

Fig 1 summarises the study workflow, including data pre-processing, quadrilateral evaluation, and trade-off analysis to address the Banking 4.0 gap between theoretical performance and operational requirements

This paper is structured as follows. The related works are reviewed in Section 2. Section 3 presents the methodology of our research. Then, we illustrate our experimental setup with different parameters, for implementation in Section 4. Section 5 describes the experimental results to provide a notable outcome from this research work. Section 6 provides comprehensive discussion on quadrilateral evaluation of EL and DL models in both the stages of our proposed framework. A component-wise ablation study is performed for our integrated pipeline validation in terms of strengths and limitations in section 7. Finally, Section 8 concludes this research’s key highlights and showed path for future research to contribute towards knowledge discovery in predictive analytics.

## 2. Related literature

Predictive analytics is pivotal in banking for data-driven decisions, skew predictions challenge model efficacy. While EL enhances accuracy and robustness in class-imbalanced scenarios, DL captures complex patterns in high-dimensional financial data but faces optimisation hurdles. This study critically examines EL and DL based literature to highlight the research gap and the importance of systematic evaluation framework for predictive analytics [1,2,23].

A recent study performed a Systematic Literature Review (SLR) based comparative performance evaluation (CPE), to enable geotechnical practitioners to select the most suitable techniques for creating a certainty and resilient ecosystem. They contributed by collectively examining four different architecture models in AI termed as Artificial Neural Network (ANN), ML; base algorithm, DL and EL in geotechnical engineering. They gathered extensive dataset of relevant literature from Web of Science (WoS) including Elsevier journal for analysis based on their approach, primary focus, year of publication, objectives, geographical distribution and results. Study demonstrated that ANN is widely utilised due to the need of real-world laboratory data in geotechnical engineering. However, EL models outperformed the rest in predictive analysis [23].

Another SLR based performance evaluation study following Kitchenham approach published recently. Authors implemented Decision Trees (DT), Random Forest (RF), Adaptive Boost (AdaBoost), and XGBoost as classifiers. The results showed XGBoost performed well on an imbalanced dataset and with B2S sampling, the performance of XGBoost increased to 0.87 from 0.50 on F1 score [2].

An approach like our study, where authors highlight the impact of imbalanced data on machine learning models for credit card fraud detection. They used sampling techniques like Random Oversampling (ROS), Random Undersampling (RUS), and SMOTE to balance the dataset and improve classifiers accuracy. XGBoost performed well on metrics like accuracy, precision, Recall and F1 score, compared to DT, and ANN on imbalanced and sampled dataset [5].

A recently published study resembles our approach, where authors implemented ML models on bank marketing dataset to evaluate the performance of classifiers and of feature engineering techniques. They evaluated the K-Nearest Neighbour (KNN), Support Vector Machine (SVM), DT, and Naïve Bayes (NB) on training accuracy and testing accuracy. Authors concluded KNN with the value of parameter k as 3 showed training accuracy of 91% and testing accuracy of 92%. They have used SMOTE to handle the problem of imbalanced dataset [15].

In a prior study, authors showed performance evaluation on implementation of Linear Regression (Ln. R), KNN, and RF classifiers. The study concluded the results by recommending RF as viable choice as classifier, with result of 96% accuracy, precision, and F1 score [16].

A study showed customer churn prediction in the banking industry and evaluated the performance of LR, SVM, RF and XGBoost. They have used accuracy, specificity, sensitivity, AUC, and F1 score as evaluative metrics. They evaluated the performance of classifiers on an imbalanced dataset and on a dataset after implementation of SMOTE as sampling technique to remove imbalance problem. Results concluded XGBoost as leading classifier on performance with 56% on F1 score with imbalanced dataset, and 61% on F1 score with SMOTE implementation on dataset [24].

A recent study conducted an SLR on ML techniques and algorithms. The research questions for this review were designed to identify different algorithms and techniques used, the performance evaluation metrics employed, and the strengths and limitations of each study. The authors searched digital libraries such as IEEE, the Association for Computing Machinery (ACM), and ScienceDirect for articles published from January 2011 to August 2021. They found fifty-seven studies after applying both inclusive and exclusive search strategies. The review revealed that classifiers such as Logistic Regression (LR), Support Vector Machine (SVM), Decision Tree (DT), AdaBoost, XGBoost, Naïve Bayes (NB), K-Nearest Neighbour (KNN), and RF are commonly used and recommended. The authors also noted that accuracy, precision, recall, F1 score, AUC and Mean Absolute Error (MAE) are often employed evaluation metrics. Additionally, approximately 60% of the studies were related to the medical field, although some research was also reviewed in the finance field [25].

Authors presented results in a SLR; based on Peterson’s systematic mapping methodology. They evaluated 9,927 research papers from seven different digital libraries focused on sampling techniques for ML. Subsequently, they selected thirty-five papers published between 2013 and 2020, covering domains such as health, finance, and engineering. The study found that ROS technique, when applied to traditional ML models, is the most prevalent, although sampling solutions involving neural networks (NN) or EL models proved superior performance [6].

Study performed a comparative evaluation of EL and DL classifiers on eleven different datasets, sourced from OpenML and Kaggle repositories. Authors implemented CatBoost, LightGBM, and XGBoost as EL classifiers. For DL models, they selected recently proposed TabNet, Neural Oblivious Decision Ensemble (NODE), Disjunctive Normal Formulas in Neural Network (DNF-Net), and 1D Convolutional Neural Network (1D-CNN). EL classifiers performed better on Root Mean Square Error, and Cross Entropy loss compared to baseline DL models. Based on these results, authors proposed using a fusion of EL and DL classifiers for tabular datasets. The study concluded that the average relative performance of the fusion model, which holds Deep Ensemble with XGBoost is best among all, with a value of 2.32% (lower value is better) [7].

In a recent study, authors contributed by conducting the largest tabular data analysis to date of publishing in 2024. They performed a comparative evaluation of nineteen algorithms, each with up to thirty hyperparameter settings, across hundred and seventy-six datasets from Open ML-CC 18 suite and Open ML Benchmarking suite. CatBoost, LightGBM, and XGBoost were included as GBDT algorithms. TabNet, MLP, and NODE are among the eleven different DL models used in this study. Baseline ML models included DT, SVM, RF, Linear model, and KNN for performance evaluation. The study assessed mean accuracy, standard deviation of normalised accuracy across folds, F1 score, and AUC as evaluative measures. The study concluded that, in many cases, strong baseline ML models or a well-tuned GBDT were sufficient to produce results. While NN are the best for a non-negligible fraction of the datasets. Study focused on the performance evaluation of NN and GBDT on tabular datasets and concluded that GBDT were much better than NN at handling skewed or heavy-tailed feature distribution and other forms of dataset with irregularities. Authors also released a benchmark guide on GitHub named ‘*Tabzilla*’, which provides a collection of studies on thirty-six of the most challenging datasets for practitioners [8].

In the realm of NN, a prior work shows that carefully searching for optimal combination of regularisation techniques in traditional MLP, referred to as ‘*regularisation cocktails*’, can produce remarkable results. Authors performed a large-scale experimental protocol for the comparative evaluation of GBDT, NN, and Transformer based models (TF) on sixty-eight different datasets from Open ML-CC 18 suite and Open ML Benchmarking suite. They also analysed the influence of hyperparameter tuning on the predictive quality of neural networks. Empirical results, based on the average ROC-AUC score with 10 outer cross- validation (CV) folds, suggest that NN are competitive with GBDT. Meanwhile, TF did not perform better than variants of traditional MLP, such as MLP with residual connection, in terms of ROC-AUC and execution time on tabular datasets [9].

In a recently published work, authors conducted a research study on enhancing credit risk prediction models to better identify qualified credit card holders, thereby improving the application process and profitability for banks. Utilising a dataset of over 40,000 records, the study aims to address the challenge of accurately predicting user credit risk. Authors employed and evaluated the performance of LightGBM, XGBoost, CatBoost, TabNet, and NN on raw data, data incorporated with Principal Component Analysis (PCA) for dimensionality reduction, and on sampled data. They have used SMOTE with edited nearest neighbours (SMOTEENN) for handling class imbalance. The results suggest improvement in F1 score, recall, precision and AUC after the implementation of classifiers on processed data through PCA and SMOTEENN. Authors concluded LightGBM as best performing model [10].

For Explainable AI (XAI), A recent publication analyses the effects of class imbalance on results of XAI tools like SHapley Additive ExPlanations (SHAP) and Local-Interpretable Model-Agnostic Explanation (LIME). An empirical study, which used sequential rank agreement (SRA) to measure the ranking stability of features generated by LIME and SHAP for each target at each default rate. Authors also measured feature importance value stability using coefficient of variations, variability stability index (VSI), and coefficient stability index (CSI) to conclude the robustness of LIME and SHAP interpretability [26].

Some recent publications suggest ‘predictive analytics for marketing using AI’, as an innovative problem of research. A related study examines the transformative role of AI in marketing, providing a comprehensive literature review of its applications and impacts. The objectives are to highlight the necessity of AI in marketing, explore its applications, and discuss its role in reshaping marketing campaigns. Authors named ethical challenges and technical constraints associated with AI adoption. Study concludes that AI enables significant innovation in marketing while suggesting future research of integrating AI with internet of things (IOT), enhancing real-time decision-making for marketing campaigns, and addressing data security concerns according to GDPR regulations [12].

A published study explores the transformative impact of AI on marketing strategies, emphasizing its ability to enhance efficiency, personalisation, and decision-making across six key areas: customer insights, performance measurement, automation, ethical implications, customer experience, and growth opportunities. The study combines a comprehensive literature review with a survey of 40 marketing professionals to provide practical insights into AI’s role in marketing. It highlights the use of AI-driven tools like predictive analytics, programmatic advertising, chatbots, and sentiment analysis, exhibiting their potential to optimise resource allocation and enhance customer engagement. Authors acknowledge ethical challenges, such as algorithmic bias and privacy concerns under GDPR regulations, and stress the need for human oversight and robust guidelines to ensure sustainable AI integration. The paper proposes future exploration of AI’s integration with emerging technologies like blockchain. This study provides a valuable roadmap for exercise AI to create impactful, data-driven, and customer-centric marketing campaigns [13].

A novel framework is presented that integrates fairness and explainability for loan approval and real-estate price prediction. Leveraging LightGBM and XGBoost, the research employs fairness-focused techniques such as *Calibrated Equalized Odds* and *Intersectional Fairness* to mitigate biases and enhance equitable decision-making. The findings highlight the importance of ethical AI in aligning with governance and regulatory standards, making it practical for real-world applications [27].

Study highlights the challenges of data privacy, bias, and regulatory compliance within banking industry. It evaluates global AI governance framework, proposes strategies and future research for ethical compliance [14].

As outlined in the related literature and summarised in Tables 2 and 3, multitudinous studies have examined the context of XAI to enhance transparency for predictive models [19–22]. Some of the studies demonstrate scenarios in which NN, GBDT, and TF perform well or fall short. For instance, GBDT models are effective with structured data but struggle with unstructured inputs. It also lacks systematic evaluation under imbalanced class distributions common in marketing campaigns. NN Models excel in high-dimensional data, their opacity and GPU dependency hinder deployment in resource-constrained banking IT-systems. TabNet model achieve state-of-the-art (SOTA) results in sequential tasks, though they can be resource intensive. Prior studies have predominantly focused on narrow technical benchmarks for GBDT, NN, or TF models, often neglecting the operational realities of banking 4.0. Predictive performance must coexist with computational frugality, regulatory transparency, and ethical governance. Therefore, our study methodology builds upon these strengths and limitations as foundational work, proposing a quadrilateral-based evaluation, which unifies discrimination, calibration, computing efficiency, and explainability into a two-stage reproducible pipeline framework. Our two-stage framework harmonises bias-aware sampling with CPU-efficient architecture, ensuring GDPR-compliant transparency, with actionable insights. By quantifying scalability through CI and demonstrating EL parity with DL models on performance, efficiency, and explainability, we bridge the critical divide between technical rigour and banking 4.0 mandate of ethical and auditable AI for decision-making [12,13,26,27].

**Table 2:** Summary of Related Work with banking dataset.

Paper	Year	Analysis Approach	Dataset	Target -Class Distributions	Results (Contributions with Limitations)
[5]	2023	CPE for credit card fraud detection in banking industry	Credit-card fraud detection	99.83:0.17	• Study includes implementation of XGBoost on imbalanced dataset, and produced better results on accuracy, precision, recall, and F1 score, compared to LR, DT, and ANN. • SMOTE sampling technique for imbalanced dataset, improved the performance of XGBoost on all discrimination-based performance metrics. Calibration, computational, and explainability based performance is missing.
[15]	2023	CPE for customer transactions in banking industry	Bank marketing dataset	Imbalanced dataset (No Distribution mentioned)	• KNN produced 92% testing accuracy and 91% training accuracy. • Study recommends the implementation of DL classifiers for comparative evaluation with KNN. • No clear statement for defining target-class distributions. Limited performance metrics.
[16]	2023	CPE for Telemarketing campaign in banking industry.	Bank marketing dataset	Balanced dataset with 5000 cases of each binary class	• Authors implemented Ln. R, KNN, and RF on the processed dataset, where RF performed better with 96% on accuracy, precision, and F1 score. (Limited performance metrics). • There is no clear statement for dataset shape, and use of feature engineering techniques.
[24]	2024	CPE for customer churn in banking industry.	Bank customer churn dataset	79.60:20.40	• Study includes the implementation of LR, SVM, RF and XGBoost. • XGBoost classifier leads with 56% on F1 score with imbalanced dataset, and 61% on F1 score with SMOTE-sampling technique. (Limited Performance metrics).

**Table 3:** Summary of Related Work with multiple datasets.

Paper	Year	Analysis Approach	Dataset	Target-Class Distributions	Results (Contributions with Limitations)
[7]	2022	CPE of GBDT with DL.	11 tabular datasets.	Multiple tabular datasets	• Authors evaluated the performance of a recently proposed DL model for tabular datasets, like TabNet, NODE, DNF-Net, and 1D-CNN with GBDT (EL). • Study includes the implementation of independent DL models and XGBoost on multiple datasets to compare the result with implementation of fusion-based models contains DL + EL classifiers. • Study suggested EL on performance, as compared to baseline DL classifiers so proposed using fusion of EL and • DL classifiers for tabular datasets. • The overall average relative performance of fusion-based models (EL + DL + XGBoost) is best among all which is 2.32. (The lower value is better) • The study included computational complexity results as well.
[8]	2024	CPE of ML, GBDT, and NN.	176 datasets	Multiple tabular datasets	• The study includes performance comparisons of 19 different ML, NN and GBDT based algorithms on 176 different datasets. • Authors concluded that GBDT are much better than NN at handling skewed or heavy-tailed feature distribution and other forms of dataset with irregularities. • This study contributes towards knowledge by ‘release of a benchmark guide’ on GitHub named ‘Tabzilla’, where a collection of study available on 36 hardest datasets, for practitioners. • No statement of handling imbalanced dataset.
[9]	2024	CPE of GBDT, NN, and TF.	68 datasets	Multiple tabular datasets	• Study includes the implementation of multiple GBDT, NN, and TF models on 68 different tabular datasets. • Authors analysed the influence of hyperparameter tuning on the predictive analysis. • Empirical results like ROC-AUC suggests that NN are competitive against GBDT. • Authors concluded that Transformer based DL are not better than variants of traditional MLP (NN). • No clear statement for handling imbalanced data.
[10]	2024	CPE of GBDT, NN, and TF.	Credit risk	98.53:1.47	• Authors implemented LightGBM, XGBoost, CatBoost, TabNet, and NN. • Study includes implementation of PCA for dimensionality reduction. • SMOTEENN is employed for data sampling in study. • Study concluded LightGBM as best performing classifier for credit risk predictive analysis.

## 3. Research methodology

The development of reliable and interpretable predictive models with scalable effectiveness, while preventing bias results, is still a critical and under-explored research gap in current scientific studies. Bank marketing campaigns struggle to identify high-impact customers from large population. Classifying them as positive responders (accepting offer), non-responders, and negative responders (declining offer), while maintaining auditable explainability presents significant challenges. Advanced predictive models, though useful, often suffer from class imbalance and opacity, leading to biased predictions and misclassifications without compliance. This leads to wasted resources and missed marketing opportunities [17–19,28].

We evaluated the proposed two-stage framework on real-world imbalanced banking data, combining ablation study to isolate component impacts with empirical benchmarks that demonstrate EL classifiers. Our framework achieved parity in both discrimination and calibration without GPU-dependency. Although GPU acceleration is known to enhance training speed, our use of CPU-based experimentation was a deliberate choice to reflect the typical computational constraints and deployment realities within banking infrastructures. This approach ensures our findings are directly relevant and practically applicable to the environments where such models are most likely to be deployed. By quantifying synthetic sampling distortion, we enable General Data Protection Regulation (GDPR) compliant audits while balancing computational frugality and infrastructural pragmatism. Our framework extends beyond explainability by embedding quantifiable auditability and traceable decision justification, ensuring each model outcome can be legally verified under GDPR’s right to explanation and Article 22 compliance. By integrating calibration reliability, bias quantification, and SHAP-based transparency, it transforms interpretability into actionable legal accountability for ethical AI governance.

In this study, sampling strategies are prioritised that address severe class imbalance while minimising distortion of the underlying data manifold. Standard SMOTE generates synthetic observations uniformly along line segments connecting minority instances. Although this approach improves class balance, it may extend the minority class into regions that are not supported by the observed data and increase overlap with the majority class. In contrast, B2S concentrates synthetic generation on minority observations located near the decision boundary. These “in-danger” samples are identified according to the class composition of their neighbourhood, and new instances are interpolated between these boundary points and their minority neighbours. Rather than oversampling all minority instances uniformly or generating additional samples in sparse regions, B2S focuses on areas where classification uncertainty is highest. This targeted sampling reduces the risk of disproportionately amplifying isolated outliers, helps preserve local class structure and feature correlations, and retains informative majority observations. Alongside tree-based models, it is capable of capturing complex feature interactions. This design can improve class discrimination and probability calibration while limiting overfitting to noisy synthetic artefacts and supporting more stable SHAP explanations under distributional shifts [2,10,19].

The theoretical suitability of B2S is particularly evident in predictive analytics for bank marketing campaigns [13,26,27]. In such datasets, positive customer responses are typically rare and often occur in regions where the behavioural characteristics of responders and non-responders overlap. Consequently, minority response cases frequently lie close to complex decision boundaries defined by demographic, financial, and historical interaction attributes. By strengthening the representation of minority observations specifically within these borderline regions, B2S improves the model’s ability to learn meaningful decision boundaries without generating unrealistic customer profiles. This boundary-focused representation is well suited to financial marketing data, where subtle differences in customer behaviour influence campaign outcomes. In this way, B2S provides a theoretically grounded mechanism for improving classification performance while preserving the structural characteristics of the observed data distribution.

Predictive modelling in bank marketing campaigns faces two key challenges. First, accurately identifying customers, which are likely to respond, help banks to distribute resources effectively. Second, the opaque nature of high-performing models undermines stakeholder trust and complicates regulatory compliance, making it harder to diagnose errors, address bias, and ensure fairness. Given the competitive nature of the banking industry, even slight improvements in predictive performance can result in substantial revenue gains, as resources are directed more effectively towards high-value customers. However, the black-box characteristic of many advanced predictive models undermines stakeholder’s confidence and complicates regulatory compliance, as decision-making processes remain concealed. This opacity can lead to difficulties in diagnosing errors, mitigating bias, and ensuring fairness across diverse customer segments.

Consequently, there is a persistent need for methodologies that not only deliver robust predictive performance but also provide transparent and interpretable insights into their internal workings, thereby enabling more informed, ethical, and effective marketing strategies. To address this, we proposed a two-stage pipeline, where stage 1 benchmarks EL and DL classifiers on raw imbalanced tabular dataset. Stage 2 applies five sampling techniques to handle the problem of imbalanced tabular dataset and re-evaluates the same EL and DL models to ensure better comparison. Furthermore, we have applied hyper-parameter optimisation search for best models to conclude the study. We have used Grid Search, Random Search, and Tree Parzen Estimates (TPE)-OPTUNA with hyperband pruning for hyper-parameter tuning. We also compute confidence interval (CI) to quantify reliability and scalability by showing how results might vary across different samples or populations (cross validation = 5). Our 5-fold cross validation used stratification technique to preserve minority class distribution across all folds in imbalanced experimentation, ensuring balanced representation in each training-validation-testing split. Finally, feature importance, and SHAP analysis, including SHAP stability offers critical insights about feature contributions and their impact, to enhance auditable explainability which is critical for GDPR-mandated transparency in banking industry.

For dataset selection, bound to GDPR and Bank’s regulatory compliance frameworks, availability of practical case studies and customer’s dataset are extremely limited. Due to intersection attacks, cyber-attacks, identity theft and many other malware attacks, banks and financial institutions are keenly reserved in sharing practical case studies or publishing any form of data for outsiders. Publicly publishing customer data in the banking industry is not only non-compliant with GDPR, Financial Conduct Authority (FCA), and Bank of England’s prudential regulatory authority (PRA) regulations but also poses significant ethical, operational, and financial risks. Adhering to strict data privacy standards, banks safeguard customer trust, protect organisational reputation, and ensure compliance with ethical and legal frameworks. With all these constraints on availability and usage of customer’s dataset, we use single dataset [4] sourced from the University of California, Irvine (UCI); an online data repository, holds customer demographics and information related to earlier marketing campaigns. It includes a binary class target variable, representing whether a customer subscribes to a product or not with 45211 instances under 16 input features with 11.69% minority class (focus of this study). As the positive class is in minority and the focus class, we need to handle the imbalance problem to avoid bias towards the majority class in training of the model [20,28,29].

To empirically assess the framework’s robustness and scalability, we implemented a comprehensive data augmentation strategy using data perturbation to enhance the volume and variability within dataset attributes for stress-testing. It introduces greater variety and diversity in input features, enabling a stress-test setting for robustness under distributional shifts. This data augmentation created an expanded dataset of 271266 samples with the same 16 input features and maintaining the target-class imbalance proportion. This six-fold increase in volume, while maintaining the original data’s statistical characteristics and class imbalance, allowed us to stress-test the model’s performance and generalisability under a larger data regime.

Our study methodology includes the implementation of data categorisation, label encoding, one-hot encoding, and sampling techniques under feature engineering to prepare the dataset for improved results. We performed statistical analysis to understand the relationship and distributional shift after sampling techniques among the features using interaction, intersectional and causal analysis in feature engineering.

Our implementation empirically highlights the impact of dataset class distribution on data-driven decision-making. Predictive ML models are sensitive to dataset distribution and bias and learns the pattern during training and will reflect that pattern in prediction. This is one of the many contributors to systemic bias, which compels ML models towards bias predictions and inference. After reviewing related literature and industry adoption of predictive models, we implemented some ensemble and DL models. We selected RF as a bagging ensemble model. Adaptive Boost (AdaBoost) and XGBoost as boosting ensemble learning models for empirical simulation. Our implementation methodology includes Single Dimensional-CNN (1D-CNN), MLP, and TabNet as DL classifiers. Sampling techniques such as Random Oversampling (ROS), Random Undersampling (RUS), Synthetic Minority Oversampling Technique (SMOTE), Adaptive Synthetic (Adasyn), and Borderline2SMOTE (B2S) are used in feature engineering to address the imbalance problem. Performance evaluation uses some relative metrics such as accuracy, precision, recall, F1 score, Area under the Curve (AUC), Confidence interval (CI), Matthew’s correlation coefficient (MCC), logarithmic loss, Brier score, training time, testing time, and memory usage to cover discrimination, calibration, and computational estimates in our research methodology. We used Brier Score and Log Loss as they are strictly proper, differentiable scoring rules that provide robust, quantitative assessments of probabilistic calibration under imbalanced data. Unlike Expected Calibration Error, Equalised Calibration Estimate or reliability diagrams, which are sensitive to binning and mainly diagnostic, these metrics ensure statistical rigour and reproducibility across models and sampling techniques. Finally, we use feature importance and SHAP analyses to improve explainability. This helps in evaluating the impact of input features on the model performance. Our research methodology includes the discussion on the scalability and applicability of the models based on multiple different metrics [27,30].

This paper evaluates the integration of sampling techniques, with explainable Artificial Intelligence (XAI) methods and predictive ML models. We intend to evaluate bias inflation tendency of different sampling techniques in second stage of our framework. This study is designed to understand and overcome inherent biases and interpretability limitations in black-box models, applied to sensitive case studies. Additionally, the study compares multiple ensemble and deep learning models across discrimination, calibration, explainability and computational measures in order to describe their trade-offs on the bank marketing task. This paper articulates both practical and theoretical implications that are directly relevant to industry.

**Fig 2:**
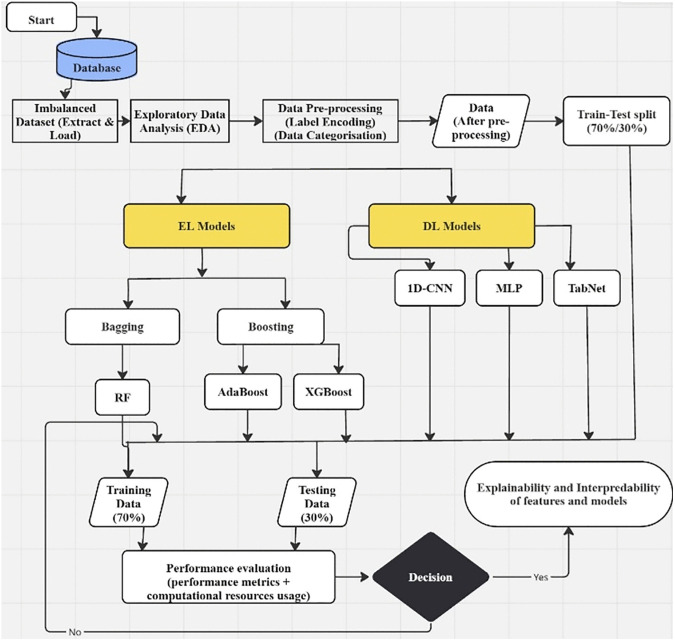
Flow chart for stage 1 of our experimental methodology with imbalanced data to quantify bias and overfitting.

**Fig 3:**
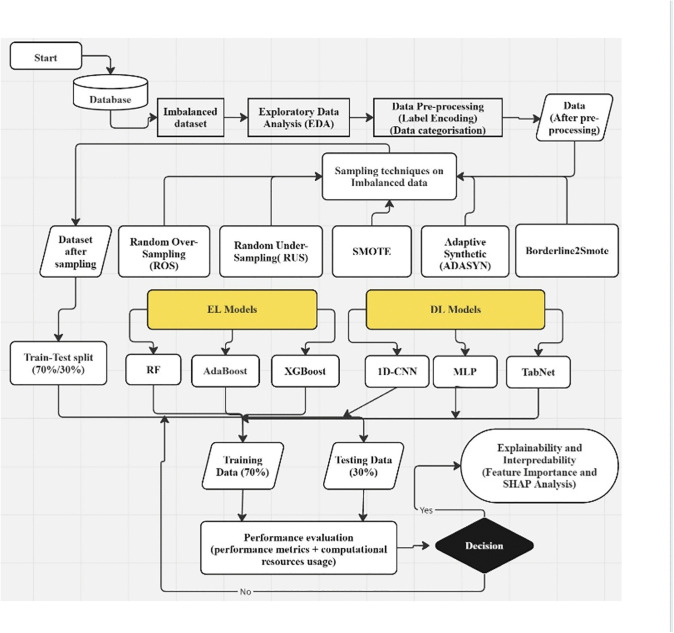
Flowchart for stage 2 of our experimental methodology with sampled data to quantify bias and overfitting.

As we aim for comparative evaluation of EL and DL models on imbalanced data and processed data with sampling techniques, so our experimental methodology consists of two stages. Figs 2 and 3 combined, illustrate overall flowchart of our experimental methodology. Fig 2 shows the experimental flow chart for EL and DL classifiers implementation on imbalanced tabular dataset. Fig 3 shows second stage, illustrating experimental flowchart, integrating black-box model with sampling techniques and XAI methods for comparative evaluation on performance, calibration, adaptability, and interpretability.

## 4. Implementation parameters

In the light of the above-mentioned related prior work, our experimental methodology is designed to advance the application of predictive analytics in bank marketing campaigns. These models support the prediction of customer response, customer behaviour, the design of effective marketing strategies, and stronger customer engagement. To keep these complex models interpretable and useful in practice, we apply the explainable AI method SHAP together with feature importance analysis. SHAP helps clarify how each feature contributes to a prediction, while feature importance highlights the variables that most strongly influence campaign outcomes. By integrating these methods, the study improves predictive performance while also promoting transparency and trust in AI systems used within the banking sector.

The proposed methodology was implemented using Python language (version:3.11.12) in Jupyter notebook, employing libraries such as scikit-learn (version:1.6.1) for EL classifiers, TensorFlow (version: 2.18.0) and Keras (version: 3.8.0) for DL architectures, and imbalance-learn (version:0.13.0) for sampling. The experiments were conducted on an Intel Core Ultra 9, personal computer with 32 GB RAM to ensure efficient processing of the dataset and smooth experimentation. The parameters in Tables 4-6) were chosen for implementation in stage 1 and 2, based on widely accepted default settings recommended in existing literature and relevant benchmarking studies [7–9].

**Table 4:** Experimental parameters for EL models used in our study.

Model	Experimental Parameters
Random Forest	We imported Random Forest classifier from ensemble of the scikit-learn library and set the ‘random state’ of machine equals to 100.
AdaBoost	We imported AdaBoost classifiers from ensemble of scikit-learn library with random state equals to 100 and implemented them on training and testing data.
XGBoost	We imported XGBoost classifiers from the machine learning library, we set the seed size at 25, n-thread’ equals to 1 and random state at 100 for implementing XGBoost on training and testing data.

**Table 5:** Experimental parameters for DL models used in our study.

Model	Experimental Parameters
1D-CNN	We used TensorFlow to import keras for CNN classifier with random state at 42. Used Adam as optimiser with parameters set as model = models_sequential, layers = Conv1D, loss = binary_crossentropy, epochs = 20, batch_size = 32.
MLP	We used scikit learn neural network to import MLP classifier with random state at 42. Parameter set at hidden_layer_sizes= (100, 50), max_iter = 200, warm_start = True.
TabNet	We used TensorFlow and imported torch for TabNet classifier with random state at 42. Used Adam as optimiser, with parameters like step_size = 50, gamma = 0.9, mask_type = entmax, max_epochs = 100, patience = 10.

**Table 6:** Experimental parameters for sampling models used in our study.

Model	Experimental Parameters
ROS	We imported Random Over Sampler from Imblearn library and applied on dataset with random state of machine equals to 0.
RUS	We imported Random Under Sampler from Imblearn library and applied on dataset with random state of machine equals to 0
SMOTE	We imported SMOTE from Imblearn library and applied it on dataset with random state of machine equals to 0.
Adasyn	We imported Adasyn from Imblearn library and applied it to a dataset with random state of machine equals to 0.
B2S	We imported B2S from Imblearn library and applied it on dataset with random state of machine equals to 0.

The imbalanced tabular bank marketing dataset underwent pre-processing stage. It includes exploratory data analysis (EDA), statistical correlation analysis, data cleaning, feature categorisation, label encoding, one-hot encoding and sampling. All the analysis performed to study that “Will a customer responds positive towards our product on a future marketing campaign”. For this, we used historic dataset of previous marketing campaigns with the outcome of that marketing campaign to train the prediction models. As historic data reflects bias distribution and imbalance proportion within target variable. Plethora of published studies used synthetic data generation techniques or sampling to address the imbalance problem but causes bias and overfitting. This study aims to get an evaluation of predictive models on imbalanced historic data in stage 1 to get the values as worst case scenario. Then in stage 2 we implemented different sampling techniques and evaluated the predictive models to quantify bias and overfitting caused by sampling techniques. Then for practical adaptability and best methodological practices we implement hyper-parameter tuning and trained the models on training split with best B2S sampling. It will limit the model to create bias towards majority and will try to train and learn. Then the imbalanced validation and testing split of historic dataset is used to evaluate the performance, computational complexity, and explainability stability of model’s decisions. We confine resampling to the training split but imbalanced testing and cross-validation to prevent data leakage, and overfitting. All the splits used stratified splitting for fair availability of classes within all variables. All the results showed in this study used a random state of 100 for EL and 42 for DL, for reproducibility of results. The shape of the post-processed dataset is shown in Table 7.

**Table 7:** Dataset for classifiers implementation after label Encoding.

Variables	Data Type	Instance values	Encoding
Y	Binary	No, yes	Label Encoding (0 and 1)
Agecat	Categorical	Young, middle-aged, aged-adults, old-age, senior citizen	Label Encoding (0–4)
Marital	Categorical	Married, Unmarried, Divorce	One-Hot Encoding
Education	Categorical	Unknown, Secondary, Tertiary, Primary	One-Hot Encoding
Default	Binary	No, yes	Label Encoding (0 and 1)
Housing	Binary	No, yes	Label Encoding (0 and 1)
Loan	Binary	No, yes	Label Encoding (0 and 1)
Contact	Categorical	Cellular, Telephone, Unknown	One-Hot Encoding
Poutcome	Categorical	Unknown, Failure, Other, Success	One-Hot Encoding
Balcat	Categorical	−8019-0, 1-1000, 1001-10000, 10001-25000, 25000+ (avg. bal in euro)	Label Encoding (0–4)
Daycat	Categorical	1-7, 8-14, 15-21, 22-28, 29-31	Label Encoding (0–4)
Monthcat	Categorical	Aug-Oct, Nov-Jan, Feb-Apr, May-July	Label Encoding (0–3)
Durationcat	Categorical	1-2, 3-4, 5-6, 7-8, 9-10, 10+ (in minutes)	Label Encoding (0–5)
Campaigncat	Categorical	1-5, 6-10, 11-15, 16-20, 21-25, 25+	Label Encoding (0–5)
Pdayscat	Categorical	1-100, 101–200, 201–300, 301 + , no contact.	One-Hot Encoding
Previouscat	Categorical	0, 1-50, 51-100, 101-150, 151-200, 200-275	Label Encoding (0–5)
Jobcat	Categorical	Unknown, Dependent, Blue-collar, White-collar, Business-owner, Retired	One-Hot Encoding

In the first stage, we implemented EL and DL classifiers on imbalanced tabular dataset to evaluate the performance on discrimination and calibration metrics. In the second stage, we first treat the imbalanced dataset with sampling techniques like ROS, RUS, SMOTE, Adasyn, and B2S during pre-processing to address the imbalanced target-class problem, by adding synthetic instances. We then implemented the same EL and DL classifiers from first experiment on sampled tabular dataset to evaluate the performance on same metrics to quantify bias inflation and overfitting. As we have got best predictive models and sampling technique with reasonable bias trade-offs, we implemented hyper-parameter tuning to best models for predictive results evaluation. At the end, we visualised results through feature importance graphs, SHAP analysis graphs, and SHAP Stability in predictions to understand the results from EL and DL models.

## 5. Results

### 5.1. Pre-processing analysis

**Fig 4:**
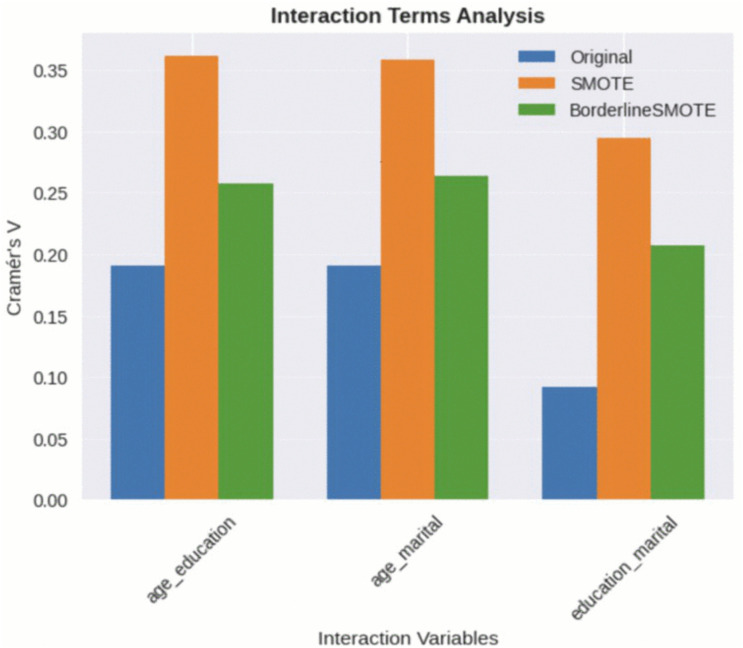
Feature correlations comparison for original data with sampling techniques, introduces artefacts.

Our statistical and exploratory data analysis includes correlation analysis, interaction terms, intersectional, and causal analysis. Our analysis reflects no missing data availability in the data. It reveals that 69% of people which responds positively to marketing campaigns previously have a profile of married, aged adults (35–60 years) with tertiary level education. We have analysed the explainability consistency, uplift in intersectional causal analysis for variables. Our correlation statistical analysis highlights ‘marital’ and ‘education’ as proxies of ‘agecat’. Our analysis indicated that duration of marketing call in minutes influences mostly converting the customer towards positive response. We calculated and compared the feature correlation metrics of the original and sampled dataset. We conducted statistical validation using Gini index, Cramér’s V. Feature associations illustrate that while B2S preserves some original relationships, it introduces artefacts by altering interaction terms and feature-target correlations compared to the original dataset. Our comprehensive evaluation demonstrates that B2S maintains feature correlations less effectively than desired, with significant changes in statistical interactions and causal feature importance patterns illustrated in Fig 4. These findings confirm that synthetic data generation requires rigorous statistical validation beyond simple performance metrics to ensure correlation preservation and artifact detection.

### 5.2. Stage 1 with Imbalanced Data

We implemented RF, AdaBoost, and XGBoost as EL classifiers. Then for DL-based CNN, we converted the tabular dataset into a one- dimensional format and reshaped the structure of training and testing datasets. For other DL classifiers like MLP, and TabNet, we adjusted key parameters such as learning rate, batch size, and the number of estimators during the training and testing process, as shown in Tables 4–6). The performance of the classifiers on imbalanced tabular dataset is presented in Table 8 using the above-mentioned discrimination and calibration metrics.

**Table 8:** Performance evaluation on imbalanced UCI dataset.

Model	Classifier	Target Variable Distribution	Accuracy	Precision	Recall	F1 Score	AUC	MCC	Log Loss	Brier Score
**(Bagging)** **EL**	Random Forest (RF)	88.31:11.69	0.86	0.54	0.37	0.44	0.85	0.39	0.77	0.09
**(Boosting) EL**	AdaBoost		0.82	0.59	0.31	0.41	0.89	0.38	0.66	0.24
	XGBoost		0.88	0.52	0.43	0.50	0.90	0.45	0.24	0.08
**Deep Learning** **(DL)**	1D-CNN		0.78	0.58	0.25	0.35	0.90	0.33	0.24	0.11
	MLP		0.80	0.51	0.43	0.46	0.89	0.41	0.27	0.09
	TabNet		0.81	0.36	0.45	0.49	0.90	0.43	0.43	0.14

As shown in Table 8 the accuracy of all classifiers is good, but since the dataset is imbalanced, discrimination and calibration estimates are not good. We need high discrimination estimates and low calibration estimates. XGBoost and TabNet are performing head-to-head on discrimination and calibration with highly imbalanced data.

This study employed three hyperparameter optimisation methods to enhance predictive performance for imbalanced bank marketing data: Grid Search, Random Search, and TPE using Optuna framework with Hyperband pruning. The TPE approach demonstrated superior optimisation efficiency for both XGBoost and TabNet architectures, with Hyperband pruning providing computational benefits through intelligent early stopping of unpromising trials. The optimised XGBoost model achieved an accuracy of 0.91 and area under curve of 0.93 with excellent probability calibration. Additionally, TabNet exhibited strong recall-oriented performance with 0.82 and balanced classification metrics despite slightly lower accuracy of 0.86 and low precision. The performance results for predictive models trained (70%), validate (15%) and tested (15%) on imbalanced data reflected the possibility of bias decision towards majority class.

### 5.3. Stage 2 with sampled data

In stage 2, multiple sampling techniques are implemented for handling imbalance data problems to explicitly evaluate the performance of same EL and DL classifiers on sampled dataset. We quantify the inflation of bias and performance using sampling techniques. Tables 9 and 10 presents the performance of EL and DL classifiers on sampled dataset across same metrics.

**Table 9:** Performance evaluation of EL on sampled UCI dataset.

Classifier	Sampling Technique	Target-class Distributions (Sampled)	Accuracy	Precision	Recall	F1 Score	AUC	MCC	Log Loss	Brier Score
**RF**	ROS	50:50	0.73	0.87	0.54	0.67	0.88	0.50	1.25	0.19
	RUS	50:50	0.82	0.81	0.83	0.82	0.89	0.64	0.53	0.13
	SMOTE	50:50	0.80	0.87	0.70	0.77	0.89	0.60	0.90	0.15
	Adasyn	50.24:49.76	0.78	0.86	0.68	0.76	0.88	0.58	0.98	0.16
	B2S	50:50	0.88	0.92	0.82	0.87	0.96	0.76	0.47	0.09
**AdaBoost**	ROS	50:50	0.82	0.81	0.82	0.82	0.89	0.63	0.58	0.19
	RUS	50:50	0.81	0.80	0.81	0.81	0.88	0.62	0.58	0.20
	Smote	50:50	0.84	0.83	0.86	0.85	0.91	0.68	0.57	0.19
	Adasyn	50.24:49.76	0.82	0.82	0.82	0.82	0.90	0.65	0.58	0.19
	B2S	50:50	0.85	0.84	0.87	0.85	0.92	0.70	0.58	0.19
**XGBoost**	ROS	50:50	0.83	0.85	0.80	0.82	0.91	0.65	0.41	0.13
	RUS	50:50	0.84	0.83	0.85	0.84	0.90	0.67	0.41	0.12
	SMOTE	50:50	0.85	0.86	0.84	0.85	0.93	0.70	0.35	0.11
	Adasyn	50.24:49.76	0.84	0.85	0.83	0.84	0.92	0.68	0.37	0.11
	B2S	50:50	**0.93**	**0.93**	**0.92**	**0.92**	**0.98**	**0.85**	**0.17**	**0.05**

**Table 10:** Performance evaluation of DL on sampled UCI dataset.

Classifier	Sampling Technique	Target-class Distributions (Sampled Training)	Accuracy	Precision	Recall	F1 Score	AUC	MCC	Log Loss	Brier Score
**1D-CNN**	ROS	50:50	0.83	0.83	0.83	0.83	0.90	0.66	0.39	0.12
	RUS	50:50	0.82	0.80	0.84	0.82	0.88	0.63	0.43	0.14
	SMOTE	50:50	0.85	0.83	0.89	0.85	0.92	0.70	0.37	0.11
	Adasyn	50.24:49.76	0.84	0.82	0.88	0.85	0.91	0.69	0.38	0.12
	B2S	50:50	0.85	0.82	0.88	0.85	0.90	0.69	0.38	0.12
**MLP**	ROS	50:50	0.77	0.84	0.66	0.74	0.88	0.55	0.73	0.18
	RUS	50:50	0.81	0.83	0.79	0.81	0.89	0.63	0.46	0.14
	SMOTE	50:50	0.82	0.85	0.78	0.81	0.91	0.64	0.47	0.14
	Adasyn	50.24:49.76	0.82	0.83	0.80	0.82	0.90	0.64	0.50	0.14
	B2S	50:50	0.76	0.86	0.62	0.72	0.88	0.54	0.75	0.19
**TabNet**	ROS	50:50	0.84	0.83	0.87	0.85	0.91	0.69	0.37	0.12
	RUS	50:50	0.62	0.61	0.65	0.63	0.67	0.24	0.66	0.23
	SMOTE	50:50	0.85	0.83	0.88	0.86	0.92	0.71	0.35	0.11
	Adasyn	50.24:49.76	0.85	0.84	0.86	0.85	0.92	0.69	0.36	0.11
	B2S	50:50	0.85	0.84	0.88	0.86	0.92	0.71	0.35	0.11

As shown in Tables 9,10, the performance of EL and DL classifiers improved across all discrimination and calibration metrics with sampling techniques. XGBoost still performed slightly better on all metrics, especially with B2S sampling, compared to the DL models. The comparatively weaker performance of TabNet with RUS is not due to implementation issues but rather reflects the intrinsic information loss caused by undersampling, which disproportionately reduces the minority class signal that TabNet’s attention mechanism depends on. In contrast, TabNet performed competitively with SMOTE and B2S, where richer feature diversity supports its representational capacity. This outcome highlights the sensitivity of deep models to data volume and class balance, reinforcing the methodological finding that sampling choice significantly affects model generalisation and interpretability. As synthetic injection and change of target-class distributions in our simulation inflates the performance. However, this methodology creates overfitting and data leakage problem in practical adaptability. For this reason, we, need to evaluate XGBoost with B2S and TabNet with B2S while using best scientific methodology practices for practical adaptability. Predictive models trained on B2S sampled training data (70%). Then stratified 5-fold validation (15%) and testing (15%) performed on imbalanced data. Table 11 shows hyper-parameter tunning results on imbalanced unseen test set for best models.

**Table 11:** Evaluative results for unseen imbalanced testing data after trained on B2S sampled data.

Models	Hyper-parameter	Target-Class Distributions	Accuracy	Precision	Recall	F1 Score	AUC	MCC	Logg Loss	Brier Score
XGBoost+ B2S	Bayesian (TPE)	88.31:11.69	0.88	0.51	0.56	0.53	0.91	0.47	0.24	0.07
TabNet + B2S	Bayesian (TPE)		0.84	0.42	0.82	0.55	0.91	0.51	0.33	0.11

We implemented EL and DL models on datasets with different target-class distributions of UCI and augmented data to validate the evaluation results of predictive models. This overcomes the limitation of single dataset with single distribution, and strengthen our marketing-agnostic study. The distinct performance profiles emerging from our analysis present meaningful strategic implications for marketing campaign deployment. There is slight improvement in evaluation of predictive models on imbalanced unseen dataset after trained on B2S sampled dataset. XGBoost demonstrates clear advantages for precision sensitive applications while minimising false positives. It optimises resource allocation, making it particularly suitable for budget constrained environments. Conversely, TabNet excels in scenarios prioritising customer reach and market penetration through its superior recall performance. We recommend selecting XGBoost when campaign costs necessitate careful targeting and precision. Whereas TabNet proves more valuable when maximising respondent identification. carries greater importance, with the optimal choice ultimately reflecting specific organisational objectives between cost efficiency and comprehensive market coverage.

### 5.4. Explainability of results

**Fig 5:**
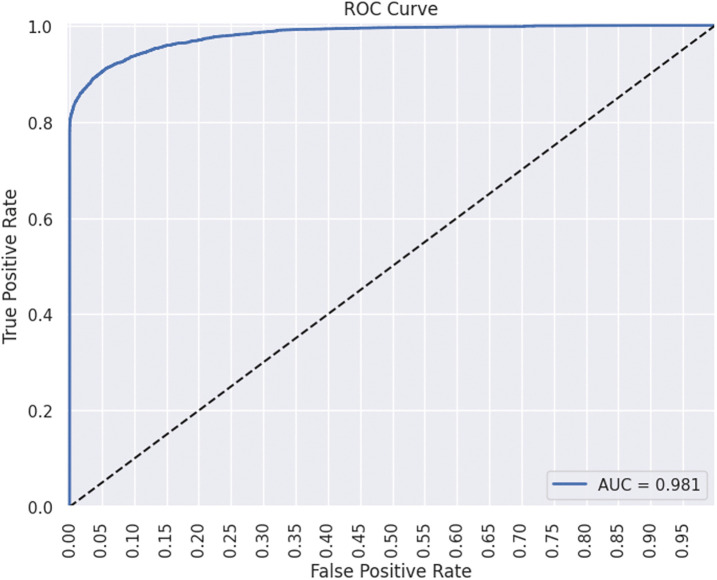
ROC-AUC plot of XGBoost classifier with B2S.

**Fig 6:**
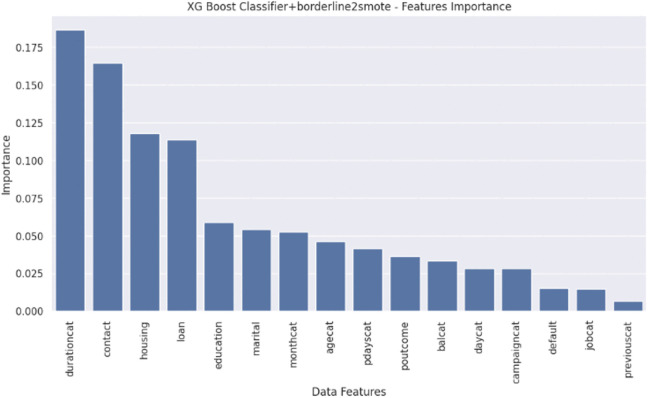
Feature importance graph of XGBoost classifier with B2S.

**Fig 7:**
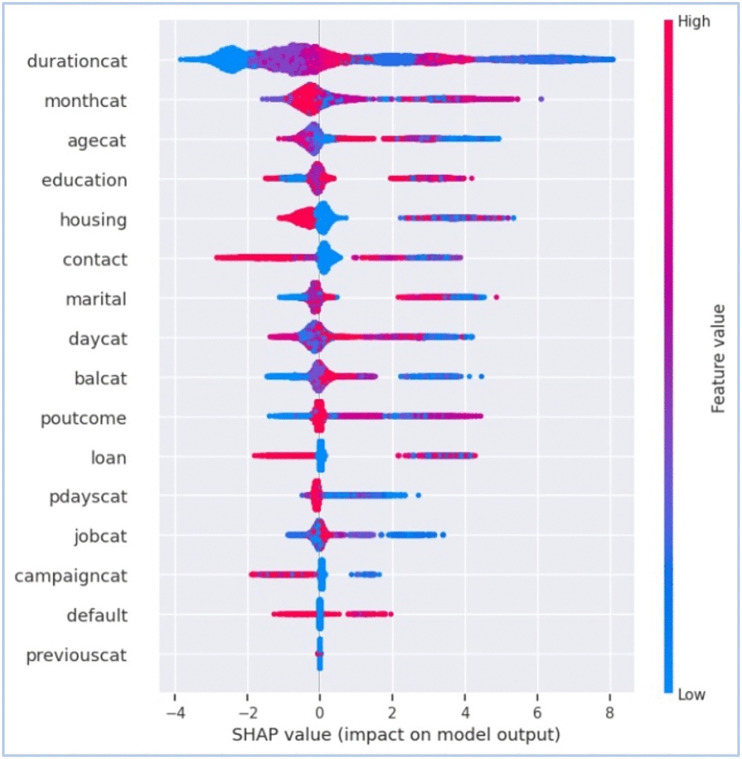
Bee-swarm plot (Global), illustration of SHAP vector behind decisions of XGBoost with B2S.

**Fig 8:**
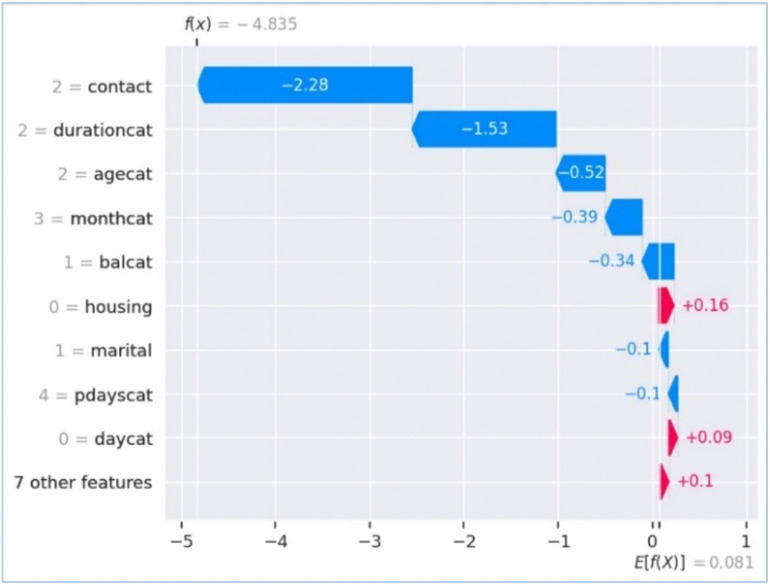
Waterfall plot (Local)), illustration of SHAP vector behind decisions of XGBoost with B2S.

Feature Importance chart, Receiver Operating Characteristics (ROC) curve, and SHAP analysis will highlight the insights for actionable guidelines. SHAP analysis uses Tree Explainer for XGBoost and Kernel Explainer for TabNet. This analysis will also explain the impact of dataset features, synthetic noise, and bias towards predictive results. The feature ‘durationcat’ proved to be the most important feature among the dataset input variables, as highlighted by both XGBoost and TabNet. Following Figs 5–8 are the results showing ROC for sensitivity and specificity, feature importance graphs, summary plots, and waterfall plots for SHAP analysis of the best EL and DL models, respectively. Fig 5 shows AUC-ROC and importance scores of input data features for XGBoost with B2S.

The ROC curve for XGBoost with B2S demonstrates exceptional performance, achieving an AUC of 0.98, indicative of near-perfect discrimination between customer classes. At a False Positive Rate (FPR) of 0.30, the model attains a True Positive Rate (TPR) of 0.80, balancing accurate identification of minority class with minimal wasted outreach. B2S enhances sensitivity to borderline cases, critical for imbalanced banking data, while the steep curve ascent underscores robustness. This performance aligns with GDPR compliance by prioritising precision in customer targeting, reducing irrelevant interventions, and ensuring ethical resource allocation. ‘durationcat’ had highest importance score. Fig 6 shows global interpretability, where ‘durationcat’ is the most influential feature to drive the predictions with highest mean absolute SHAP value. It represents SHAP waterfall plot for local interpretability by illustrating the step-by-step contribution of each feature to a single prediction. In this example, features such as ‘contact’ and ‘durationcat’ strongly push the prediction downward, leading to a negative final score of −4.835. This transparent breakdown helps us understand not only which features matter but also their directional impact on individual decisions, an essential aspect of fair and explainable AI in banking

The Fig 5 and 6 are representing results for XGBoost with B2S model for prediction. Fig 5 explains the importance of each input feature while showing the percentage score of each input variable to the overall cumulative impact of all features on the prediction result by the model. Fig 6 provides the SHAP analysis for better explainability about the impact of different input features on the prediction of the model. It shows the overall importance of features and how they influence the XGBoost with B2S model’s output. SHAP values on x-axis indicates the impact of each feature on the model’s prediction. Negative values push predictions towards the negative class (i.e., No or 0). Positive values push predictions towards the positive class (i.e., Yes or 1). List of features in descending order of importance are showed on y-axis. Features at the top contributes the most to the model’s prediction. Blue represents low feature values and red shows high feature values among the colour gradient of the plot.

The SHAP analysis and feature importance scores for XGBoost with B2S reveals that ‘durationcat’ and ‘monthcat’ are the most influential feature, with high SHAP values driving predictions towards target minority class. It revealed that ‘durationcat’ and ‘monthcat’ cumulatively drove 72% of the model predictions, enabling granular auditability of conversion outcomes. This transparency directly addresses GDPR’s mandate of ‘right to explanation’ by isolating actionable decision drivers for stakeholder review and regulatory compliance. Features like ‘contact’ and ‘housing’ also significantly change model decisions, while ‘default’ and ‘jobcat’ show minimal influence. This alignment between XGBooost with B2S intrinsic feature importance and SHAP’s local/global explanations underscores the model’s transparency. The result suggests prioritising customer interactions during ‘monthcat’=(November-January) with ‘durationcat’= (7–10 + min) has higher conversion rate as shown in Fig 7.

Fig 8 shows waterfall type of visualisation. It explains how individual features contribute to a specific model prediction for one data point. The average model prediction is E[f(x)] =0.081. The base value is f (x)= −4.83 in the XGBoost with B2S prediction model. The feature ‘contact’ significantly reduces the prediction by showing −2.28 SHAP value in overall model’s prediction. ‘agecat’ adds further negative influence and strongly pushes the prediction towards the negative class (i.e., No or 0). ‘balcat’ and ‘daycat’ push the prediction towards positive class (i.e., Yes or 1), but their impact is minimal compared to the negative influence features.

The results for calculating feature importance under simple feature importance plot and SHAP analysis plots are slightly different in feature rankings and its values under the same experiment conditions and dataset due to different calculation methodologies. XGBoost simple feature importance is based on gain/split count/ cover in trees. SHAP feature importance calculates shapley values following game theory framework. SHAP feature importance methodology is less biased towards high-cardinality features, whereas XGBoost simple feature importance plots can be biased towards features with more splits.

**Fig 9:**
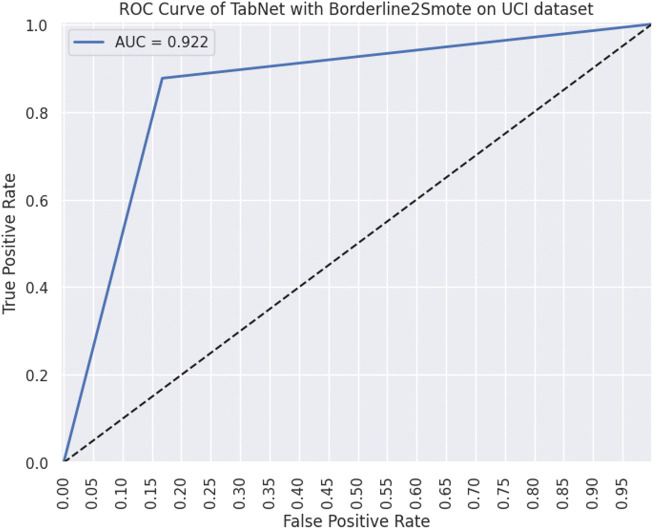
ROC-AUC plot for TabNet classifier.

**Fig 10:**
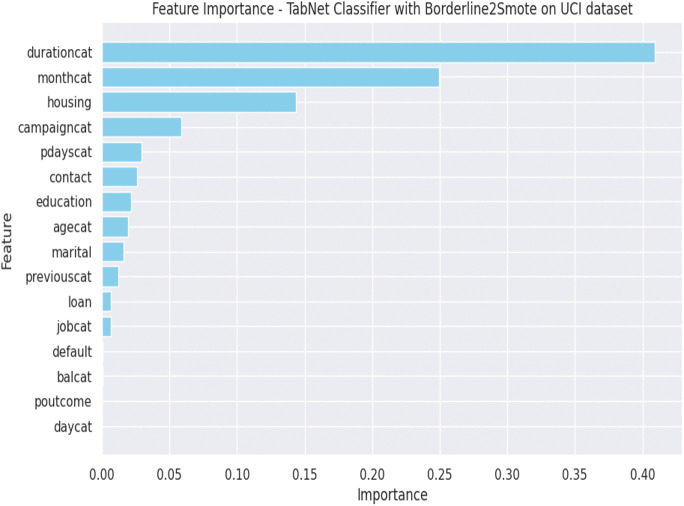
Feature importance graph for TabNet classifier.

**Fig 11:**
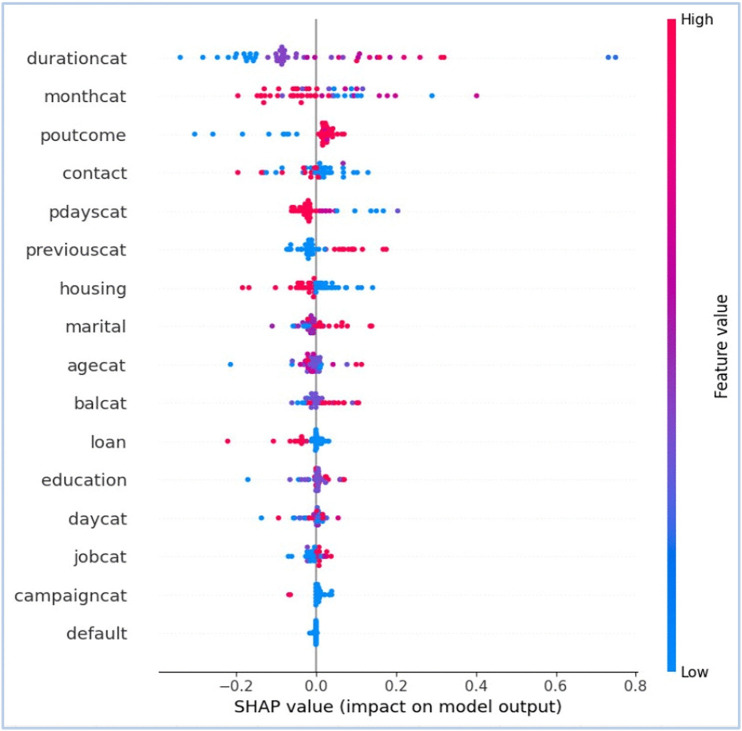
Bee-swarm plot (Global), illustrating the SHAP vectors behind decisions by TabNet.

**Fig 12:**
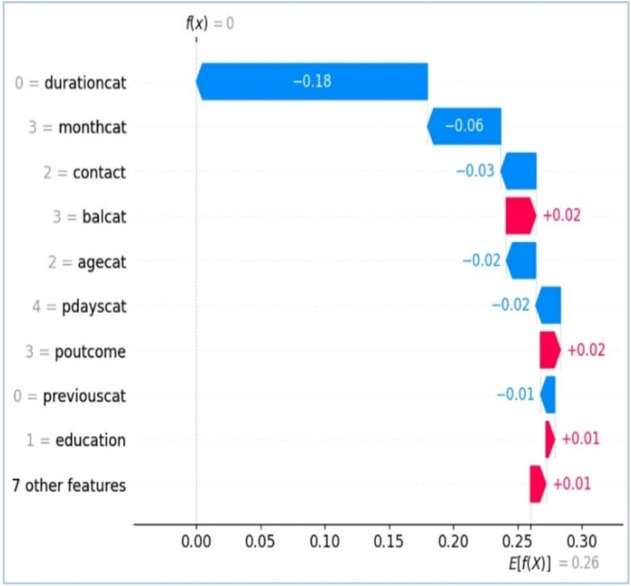
Waterfall plot (Local), illustrating the SHAP vectors behind decisions by TabNet.

Following Figs 9–12 are representing results for TabNet with B2S model for prediction. Fig 9 shows ROC curve and Fig 10 illustrates feature importance for TabNet with B2S.

The ROC curve for TabNet with B2S on the UCI dataset achieves a strong AUC, showing robust discrimination between customer classes. The model balances a high TPR with controlled FPR, ensuring reliable identification of subscribers while minimising misdirected marketing efforts. B2S enhances sensitivity to borderline minority class, critical for imbalanced banking data, while TabNet attention mechanism provides inherent interpretability. This performance underscores TabNet suitability for ethical AI applications in marketing, where transparency and precision in customer targeting are paramount.

Fig 11 provides the SHAP analysis for better global explainability about the impact of different input features on the prediction. It is known as summary plot, where x-axis shows SHAP values, standing for impact of each feature on the model’s output. Negative SHAP values push the prediction towards the negative class (i.e., No or 0), while positive values push it towards the positive class (i.e., Yes or 1). Y-axis shows input features in order of importance. The most important feature is at the top (i.e., ‘durationcat’). The feature ‘durationcat’ has the most significant impact, with higher values during the prediction towards the positive class. ‘monthcat’, ‘poutcome’, and ‘contact’ are the prominent features show substantial effect. Features like ‘default’ and ‘Campaigncat’ have less influence on the TabNet with B2S’s prediction. Fig 12 with waterfall plot for SHAP analysis visualises the contribution of the top features for predicting the outcome of particular instance in a marketing campaign. Blue bars show features pushing the prediction towards the negative class, while red bars indicate those pushing it towards the positive class. ‘durationcat’ has the largest negative effect (i.e., −0.18) pulling the prediction towards the negative class. Features like ‘balcat’ and ‘pdayscat’ contributes positively, but with smaller magnitudes. The base value for the model is f (x)=0 in the waterfall plot. It serves as the starting point for the prediction. The plot accumulates feature contribution step by step from the base value to the final model output. The net prediction value of TabNet with B2S model is E[f(x)] = 0.26.

The results of our experimentation explain the comparative performance of EL and DL models on the imbalanced dataset and on a sampled dataset, using multiple performance metrics. Implementation on the augmented dataset yielded consistent results with those from the original UCI data. XGBoost with B2S maintained its leading performance, with the AUC-ROC shifted from 0.98 to 0.93 and the F1-score stabilising at 0.90. Crucially, the trend of relative performance hierarchy between models remained unchanged, and the key explanatory features identified by SHAP analysis (e.g., ‘durationcat’, ‘monthcat’) retained their dominance. This demonstrates the framework’s stability and its capacity to leverage larger data volumes for potentially enhanced, yet consistent, predictive accuracy and explainability. The ‘durationcat’ feature was retained as it represents a post-campaign behavioural attribute, included solely for benchmarking and interpretability consistency with prior studies on the same UCI dataset. To prevent data leakage in real-world deployment, we explicitly suggest that ‘durationcat’ should be excluded from prospective inference models and used only for retrospective analysis and feature explainability validation. B2S synthetic sampling produces more stability in explanation as compared to SMOTE and original imbalanced dataset. Overall, 0.64 stability index score in explainability, while comparing to SMOTE which is 0.48 for UCI dataset.

## 6. Discussion

In this work, we exhibit a judicious combination of sampling, EL and XAI, which can reconcile the often-conflicting demands of predictive performance, computational efficiency, and regulatory compliance in banking 4.0 marketing applications. By benchmarking both traditional EL and DL on imbalanced and synthetically balanced bank-marketing dataset, we quantified not only discrimination and calibration but also CPU-only training latency, memory footprint, and SHAP-based feature transparency. This study confirms that traditional EL classifiers, particularly XGBoost, not only match but significantly outperform DL models on imbalanced bank marketing dataset and on sampled dataset, achieving superior discrimination, calibration, computational estimates. SHAP analysis enhance the explainability, while integrating with XGBoost. XGBoost probabilities are reliable (Brier = 0.05 and 0.07 in sampled testing split and imbalanced testing split, respectively) and proved as faster and lighter in computing. B2S maximises performance without sacrificing interpretability of features. Our findings advocate, integrated framework (XGBoost+B2S+SHAP) as the preferred solution for GDPR compliant banking applications, balancing performance, efficiency, and transparency, while DL models are still niche tools for scenarios with complexity and large dataset with high computational resources.

### 6.1. Adaptability implications

The results of this study illustrate the effectiveness of EL and DL models after pre-processing imbalanced data through feature engineering, including various sampling techniques, label encoding and data categorisation for predictive analysis in the banking sector. Building on the results presented, this section examines the implications of our findings, particularly regarding the practical application of EL and DL models for marketing campaigns in the banking industry. It discusses the observed performance trends, the challenges of handling imbalanced datasets, and the trade-offs between model discrimination, calibration, computational efficiency, and interpretability. Our results offer insights into the best practices for deploying these models in real-world bank settings. At the end of our discussion, we will illustrate other constraints to face with, which produce constraints in our approach’s overall scalability and generalisability [19,22].

Although XGBoost outperformed other EL and DL models overall on the imbalanced and sampled tabular datasets, particularly in terms of F1 score and mcc. TabNet with smote and B2S also delivered competitive results in these metrics. However, the practical feasibility of these models also hinges on their computational complexity. The computational resource usage report, summarised in Table 7–9 provides a detailed comparison of classifier’s accuracy, training time (in seconds), testing time (in seconds), memory usage (in MB), and confidence interval (95%) for both the imbalanced and sampled datasets. These metrics highlight the computational cost associated with model’s predictions, offering valuable insights into their efficiency and adaptability for practical deployment in real-world marketing campaigns. We also set cross validation score equals to 5 (cv = 5) for all classifiers to calculate CI. The following Table 12 shows computation complexity for EL and DL models on imbalanced dataset.

**Table 12:** Computational resources usage report for classifiers on imbalanced UCI dataset.

Model	Classifier	Accuracy	Training Time (s)	Testing Time (s)	Memory usage (MB)	Confidence Interval (%)
**(Bagging) EL**	RF	0.89	1.06	0.06	177.76	0.886-0.894
**(Boosting) EL**	AdaBoost	0.89	1.5	0.14	361.57	0.892-0.899
	XGBoost	0.90	5.27	0.15	165.62	0.893-0.902
**Deep Learning**	1D-CNN	0.89	83.44	1.51	875.03	0.893-0.897
	MLP	0.89	134.71	0.07	315.55	0.889-0.891
	TabNet	0.90	188.12	0.46	730.72	0.892-0.904

Above Table 12 shows that overall, XGBoost appears as the most balanced model in terms of performance and computational efficiency on imbalanced dataset, making it highly suitable for practical deployment in resource-constrained environments. The results indicate that XGBoost and TabNet achieved the highest classifier accuracy of 0.90, followed by other models. XGBoost stands out for its optimal combination of high accuracy and low computational cost with a training time of 5.27 seconds and testing time of 0.15 seconds, and moderate memory usage of 165.62 MB. In contrast, TabNet and other DL models, while competitive, may require added resources, limiting their feasibility for real-time applications with imbalanced dataset. TabNet, while equally accurate, exhibited significantly higher training time of 188.12 seconds and memory usage of 730.72 MB, suggesting a trade-off between interpretability and computational complexity [19,22].

The following Tables 13 and 14 shows computation complexity for EL and DL classifiers with different sampling techniques.

**Table 13:** Computational resources usage report for EL classifiers on sampled UCI dataset.

Classifier	Sampling Technique	Accuracy	Training Time (s)	Testing Time (s)	Memory Usage (MB)	Confidence Interval (%)
**RF**	ROS	0.73	5.21	0.47	524.75	0.951-0.957
	RUS	0.82	0.82	0.06	648.75	0.818-0.838
	SMOTE	0.80	4.56	0.50	798.38	0.898-0.943
	Adasyn	0.78	4.51	0.47	936.57	0.878-0.903
	B2S	0.88	6.57	0.45	1047.68	0.876-0.994
**AdaBoost**	ROS	0.82	1.24	0.08	372.64	0.815-0.823
	RUS	0.81	0.28	0.02	388.25	0.820-0.823
	SMOTE	0.84	1.85	0.29	419.8	0.815-0.862
	Adasyn	0.82	1.33	0.09	436.96	0.891-0.897
	B2S	0.85	1.69	0.08	465.72	0.826-0.870
**XGBoost**	ROS	0.83	0.84	0.07	346.86	0.895-0.901
	RUS	0.84	0.36	0.02	359.60	0.823-0.857
	SMOTE	0.85	1.0	0.07	387.71	0.865-0.921
	Adasyn	0.84	1.9	0.11	413.28	0.849-0.897
	B2S	**0.93**	**0.9**	**0.06**	**452.69**	**0.836-0.915**

**Table 14:** Computational resources usage report for DL classifiers on sampled UCI dataset.

Classifier	Sampling Technique	Accuracy	Training Time (s)	Testing Time (s)	Memory Usage (MB)	Confidence Interval (%)
**1D-CNN**	ROS	0.83	115.49	1.32	937.69	0.834-0.842
	RUS	0.81	19.44	0.23	1093.06	0.798-0.834
	SMOTE	0.85	120.0	1.33	1207.25	0.852-0.862
	Adasyn	0.84	132.45	1.33	1311.13	0.835-0.849
	B2S	0.84	114.39	2.64	1417.34	0.841-0.853
**MLP**	ROS	0.77	199.73	0.13	766.7	0.899-0.909
	RUS	0.81	19.21	0.05	945.36	0.797-0.824
	SMOTE	0.82	84.14	0.08	1017.71	0.859-0.915
	Adasyn	0.82	128.8	0.07	1035.49	0.834-0.879
	B2S	0.76	65.53	0.09	1049.81	0.892-0.926
**TabNet**	ROS	0.84	310.31	0.61	1351.68	0.796-0.916
	RUS	0.62	31.06	0.11	1273.07	0.713-0.807
	SMOTE	0.85	322.09	0.65	1502.21	0.821-0.909
	Adasyn	0.85	321.81	0.62	1520.15	0.7983-0.845
	B2S	0.85	321.90	0.63	1536.95	0.801-0.930

The computational complexity evaluation of classifiers on sampled dataset highlights notable trends in the performance of EL and DL classifiers with B2S, achieved the highest accuracy of 0.93 while keeping low computational complexity, with training time of 0.9 seconds, testing time of 0.06 seconds, and memory usage of 452.69 MB. Its combination of robust performance and low resource requirement makes it particularly suitable for deployment in real-world applications. Contrarily, TabNet with B2S, achieved comparable accuracy of 0.85, but incurred substantial computational cost with a training time of 321.90 seconds, testing time of 0.63, and memory usage of 1536.95 MB.

Even though TabNet with SMOTE, TabNet with B2S, and XGBoost with B2S have almost overlap confidence intervals, therefore the choice between selection of models should be guided by additional factors such as computational efficiency, robustness, scalability, and practical integration requirements. XGBoost requires minimal resources for data preprocessing, handling missing values and categorise data effectively with hard splits. XGBoost and TabNet, are both well scalable, but TabNet is slower compared to XGBoost in production environment due to its attention mechanism and intensive computation. MLP demands extensive data preprocessing and feature scaling with substantial computational resources and may require specialised hardware. With strong pattern recognition ability for complex relationships in large datasets, MLP is prone to overfitting and lacks inherent interpretability. Avoiding overfitting while enhancing interpretability is still a persistent challenge in selecting effective parameters across all models, illustrating the need for further research to improve their generalisability.

Due to Exhaustive search space for UCI and augmented data, computationally Grid search and Random search optimisation techniques are found costly in our simulation. TPE with early stopping using hyperband pruning reflected better signals in computational cost estimation compared to rest. The CI for XGBoost and TabNet with TPE on UCI and augmented data illustrated relative cross-sectional ranges for imbalanced stratified validation, after trained with B2S sampled data. Overall, 91–92% range for XGBoost with TPE on UCI dataset, whereas 79–90% range for TabNet with TPE on UCI data.

### 6.2. SHAP analysis audit

SHAP overcomes the model interpretability problem effectively and increases explainability in EL and DL complex models as mentioned in our implementation and results section. The force plot and waterfall plot for SHAP analysis increase the transparency and help the stakeholders to understand the steps of model up to its prediction, as well as potential biases. It also suggests the areas of improvement within distinct phases of overall marketing strategy. SHAP offers high precision by calculating the contribution of each feature to every prediction. It provides a global and local view of feature importance with consistency, detail visualisation and feature interaction. The key takeaway by SHAP analysis of XGBoost with B2S and TabNet with B2S is that ‘durationcat’ and ‘monthcat’ are critical drivers for prediction, whereas some other features cumulatively push the prediction slightly in the positive direction, but the dominant forces lead to a negative prediction. In our research paper, the waterfall model plays a key role in providing detailed and transparent insights. It illustrates exactly how each feature influences a specific prediction by showing their individual contributions step by step. This visual breakdown helps us understand which features push a prediction towards a positive or negative outcome and by how much. For example, in our analysis, we found that ‘durationcat’ had noticeable negative impact on predictions, while ‘balcat’ and ‘daycat’ had smaller but positive effects. These insights are valuable because they help us see which features have the most influence on the model’s decisions, supporting transparency and fairness in our predictive framework. While interpreting SHAP analysis results of the above-mentioned models, we suggest focusing on optimising call duration (durationcat) and prioritising favourable months (monthcat) for outreach.

In the light of our results, sampling techniques such as SMOTE, Adasyn, and B2S enhanced the performance of all EL and DL models across all metrics. It is evident that tuning parameters and selection criteria for sampling techniques greatly depends on research questions and the importance of class instance in target feature. SHAP analysis highlights bias inflation and fairness concerns with SMOTE, especially for ‘seniors’ in ‘agecat’. B2S mitigates this while reducing bias inflation by 31.4%, alongside improved recall and reduced overfitting. SMOTE disproportionately penalised demographic features. This enlightens the need for synthetic data techniques to not only balance classes but also mitigate bias for fairness and consistent explanation across all sub-groups of variables. In contrast, B2S focuses on borderline instances reducing synthetic noise, aligning with GDPR fairness mandate, while supporting performance and computational pragmatism. It offers better results along with XGBoost and is comparable well with TabNet. XGBoost may be more sensitive to borderline synthetic samples, amplifying their impact during decision tree splits. TabNet regularisation and feature masks can suppress overfitting to synthetic samples but need fine tuning for less variability in B2S case.

The SHAP based explanations offer a quantitative, auditable foundation for understanding complex model decisions, but we recognise that SHAP alone may not fully meet GDPR’s broader expectations for decision interpretability in all deployment settings. Accordingly, our framework positions SHAP as the analytic core while recommending supplementary rule-based or surrogate models to provide human-readable decision logic, ensuring alignment with GDPR’s *right to explanation* and organisational accountability requirements.

The comparative analysis confirms XGBoost combined with B2S and SHAP as the optimal approach for imbalanced banking data, excelling across discrimination, calibration, computational efficiency, and auditable explainability. Our empirical results indicate improve recall (0.43 to 0.92) due to class distribution shifts after sampling and with balanced proportion. SHAP analysis highlighting that optimising ‘durationcat’ and ‘monthcat’ boosts conversions prediction by 23–32%. These two features mainly contribute towards model prediction. B2S further reduces synthetic noise by 25%, reducing overfitting as compared to SMOTE bias inflation. It also enhances XGBoost F1 to 0.92 and AUC to 0.98. XGBoost superior generalisability (CI = 83.6–91.5%) on UCI and (CI = 81.2–89%) on augmented data. It provides faster training, and marginally better calibration than TabNet, support real-time, ethical deployment in resource-constrained banks.

Our global and local interpretability analyses, shown in SHAP plots, provides actionable insights into the model’s reasoning in decision-making process. Globally, ‘durationcat’ and ‘monthcat’ emerge as the most influential features driving positive campaign conversions across both XGBoost and TabNet, underscoring their consistent predictive power. The variable ‘durationcat’ indeed exhibits the highest SHAP contribution across models, this does not imply that other features lack predictive value. Rather, it reflects the strong causal linkage between call duration and customer conversion, which is well-documented in marketing analytics literature. Our SHAP and feature importance analyses further reveal that secondary features such as ‘monthcat’, ‘contact’, and ‘poutcome’ also contribute meaningfully by modulating the predictive context particularly in interaction with durationcat, to refine classification boundaries. Thus, the framework captures multi-feature interplay that enhances both interpretability and compliance.. Locally, the waterfall plots reveal how these features, along with ‘contact’ and ‘balcat’, contribute positively or negatively to individual predictions, offering fine-grained transparency into each decision. These findings confirm that our integrated quadrilateral framework not only enhances predictive accuracy but also delivers explainable and actionable insights for strategic marketing decisions, fulfilling both ethical AI and regulatory mandates in banking 4.0. Nonetheless, a limitation of our preliminary experimentation is the absence of comprehensive advanced hyperparameter optimisation and bias-aware techniques, which constrains the fairness of comparisons between XGBoost-B2S-SHAP and TabNet-B2S-SHAP. Additionally, our study does not benchmark these models against the latest State-of-the-Art (SOTA) techniques from prior work. Furthermore, while our study offers a thorough empirical evaluation using established algorithms and techniques, it does not propose novel theoretical frameworks that could provide deeper insights or fresh perspectives on model performance. As a result, the absence of this theoretical foundation limits the broader interpretability and generalisability of our findings. Addressing this gap through future work could help us develop a more principled approach and new theoretical contributions to support the empirical results and guide their application in the context of banking marketing analytics.

## 7. Component-wise ablation study for framework validation

The adoption of ML in imbalanced tabular marketing dataset has historically prioritised discrimination metrics, and SMOTE, often neglecting calibration, computational efficiency, and explainability. While prior works benchmarked standalone techniques, they inadequately address the banking 4.0 mandate for holistic, auditable, and resource-efficient solutions. To bridge this, we conduct a systematic ablation study to dissect the contributions and limitations of each component in our framework. Table 15 quantifies the impact of these components on discrimination, calibration, and computational costs. Algorithm 1 formalises our workflow for reproducibility.

**Table 15:** Component-Wise Ablation Analysis.

Model	F1 Score	MCC	AUC	Brier Score	Train Time
**XGBoost (Imbalance train/test data)**	0.50	0.45	0.90	0.08	5.27
**SMOTE+ XGBoost**	0.85	0.70	0.93	0.11	1.0
**B2S+XGBoost**	**0.92**	**0.85**	**0.98**	**0.05**	**0.9**
**TabNet (Imbalance train/test data)**	0.49	0.43	0.90	0.14	188.12
**SMOTE + TabNet**	0.86	0.71	0.92	0.11	322.09
**B2S + TabNet**	0.86	0.71	0.92	0.11	321.90
**XGBoost (B2S Trained/ Imbalanced Test)**	0.53	0.47	0.91	0.07	3.68
**TabNet (B2S Trained/ Imbalanced Test)**	0.55	0.51	0.91	0.11	132.26

Our empirical results demonstrate that XGBoost-B2S, along with post-hoc SHAP explainability in a two-stage framework, produced better results without any hyper-parameter tuning. It performed slightly better than TabNet-B2S in both discrimination and calibration, while staying computationally frugal. SHAP analysis added value as post-hoc explainability in our framework. Table 16. Shows algorithmic workflow to ensure reproducibility and transparency to build an integrated approach.

**Table 16:** Algorithmic workflow.

Algorithm 1 Pseudocode for XGBoost-B2S-SHAP
**Input:** Training set D =${\left\{({\text{x}}_{\text{i}},\;{\text{y}}_{\text{i}})\right\}}_{\text{i}=\text{1}-\text{N}}$ , where ${\text{x}}_{\text{i}}$ ⋿ $R^{d}$, ${\text{y}}_{\text{i}}$ ⋿ {0,1}, T: Number of decision trees, max_depth: Maximum tree depth, ${\text{k}}_{\text{neighbour}}$, ${\text{m}}_{\text{neighbour}}$: B2S parameters, M: Number of instances to explain. **Output:** Trained XGBoost Model as model and SHAP values as SHAP_values ⋿ $R^{M*d}$ **Require:** D is Imbalanced data (minority class y = 1), ${\text{k}}_{\text{neighbour}}$ ≤ ∣ ${\text{D}}_{\text{min}}$∣, where ${\text{D}}_{\text{min}}$ is the minority class subset. **Ensure:** Synthetic samples preserve minority class structure (Synthetic Noise), and SHAP values quantify feature importance for M instances.
1: **Procedure** XGBOOST-B2S-SHAP (D, T, max_depth, ${\text{k}}_{\text{neighbour}}$, ${\text{m}}_{\text{neighbour}}$, M) 2: **Step 1:** B2S SDG 3: ${\text{D}}_{\text{min}}$←{(x,y) ∈ D ∣y = 1} (Minority class subset) 4: ${\text{N}}_{\text{min}}$ ← ∣ ${\text{D}}_{\text{min}}$∣ 5: **for each** x ∈ ${\text{D}}_{\text{min}}$**do** 6: Find ${\text{m}}_{\text{neighbour}}$ nearest neighbours in D 7: **if** ≥ ⌈${\text{m}}_{\text{neighbour}}$/2⌉neighbors are majority **then** 8: Mark (x) as “in danger” 9: **end for** 10: **for each** “in danger” instance (x) **do** 11: Find ${\text{k}}_{\text{neighbour}}$ nearest neighbours in ${\text{D}}_{\text{min}}$ 12: Synthesize ${\text{x}}_{\text{new}}$ via interpolation: 13: ${\text{x}}_{\text{new}}$ ← x + λ⋅(${\text{x}}_{\text{neighbour}}$− x), λ∼U(0,1) 14: Add ${\text{x}}_{\text{new}}$ to ${\text{D}}_{\text{synth}}$ 15: **end for** 16: D′ ← D ∪ ${\text{D}}_{\text{synth}}$ (Sampled dataset) 17: **Step 2**: Train XGBoost on D′ 18: Initialize model with T, max_depth, objective = “ binary: logistic” 19: **for** t ← 1 **to** T **do** 20: Build tree t: 21: At each node, split on feature j maximising gain: 22: Complexity: O (T ⋅ d$\;.{∥\text{x}∥}_{\text{0}}$ ⋅ log $N^{\prime }$) 23: **end for** 24: **Step 3:** Compute SHAP Values (Post-Hoc Explainability) 25: Initialise explainer←TreeSHAP(model) 26: **for** i ← 1 **to** M **do** 27: SHAP_values[i]←explainer. shap_values (${\text{x}}_{\text{i}}$) 28: Complexity: O (T ⋅ L⋅$d^{\text{2}}$) per instance 29: **end for** 30: **return** model, SHAP_values 31: **end procedure**

Algorithm 1 encapsulates the integration of B2S oversampling, XGBoost based training and predictive model, and SHAP-based interpretability. This structured representation aligns with our two core contributions:

1. Quadrilateral evaluation under 2-stage framework (imbalanced and sampled).
2. Trade-offs between sampling and interpretability.

The pseudocode adheres to reproducibility standards, explicitly defining inputs, outputs, pre/post conditions, and computational complexity annotations. This study enhances transparency, and highlights bias inflation and class overlap as limitations, with precision-recall trade-offs in skewed datasets. Our modular framework mitigates this by decoupling oversampling, training, and explanation phases, enabling adjustments like ${𝐤}_{𝐧𝐞𝐢𝐠𝐡𝐛𝐨𝐮𝐫}$.

These simulation results indicate trade-offs between performance, fairness and explainability for data-driven predictions. Societal bias distribution, synthetic sampling to cater distributional imbalance costing fairness and consistent reasoning for explainability of predictive decisions. This inflated performance but bias costing the ethical AI principles. Synthetic data generation solves the imbalance problem for training, but weaken the fairness and causes systemic bias for predictive models on unseen data. Some recent studies suggest deep architecture like Generative Adversarial Networks (GANs) and their variants for synthetic data generation. This benchmark comparative study and empirical-based ablation of our framework proved the effectiveness of XGBoost and TabNet as classifiers for our dataset. We can use hybrid logic with B2S (SDG by GANs or LLM architectures) while integrating fairness-aware techniques for our case study in future. We are aiming for a hybrid model which can integrate attention-based interpretability of transformers with high performance EL alongside advanced hyper-parameter searches to accelerate effective resource allocation. Also using multi-domain dataset from healthcare, insurance, human resource hiring. Our proposed framework provides the agility for continuous iterative changes in terms of models and techniques to achieve the goal of “Holistic Approach for Predictive Modelling of Marketing Campaigns in Banking 4.0” [14].

## 8. Conclusion and Future work

This study validates a two-stage evaluation framework for imbalanced banking data that brings together discrimination, calibration, efficiency, and explainability in a single assessment. XGBoost combined with B2S sampling and SHAP analysis shows clear improvements then rest, supported by confidence intervals. It provides meaningful correlation and causal insights into customer behaviour during marketing campaigns. The results also show reduced synthetic noise and lower bias inflation, although some inconsistencies remain, particularly for sensitive demographic groups. B2S performs better than the other sampling methods, yet it still introduces bias that can influence model behaviour and create systemic patterns in predictions. XGBoost emerges as a practical option for Banking 4.0 because it balances performance with transparency and efficient use of computational resources. These findings should be viewed as preliminary due to the study’s limited scope, but it offers a useful benchmark for future research. The work demonstrates how advanced ML models can support ethical and responsible AI in marketing by providing a structured way to compare models and quantify synthetic bias. The results also show that oversampling can amplify existing societal biases and generate inconsistent explanations, which may undermine fairness for demographic sub-groups. This highlights the continuing need for approaches that preserve fairness while ensuring reliable, consistent and transparent model reasoning.

In future work, we will expand this framework to establish fairness-aware and explainable predictive analytics to support data-driven decision-making. We plan to incorporate many-objective optimisation techniques and multi-criteria decision-making methods, so that utility, fairness and explanation stability can be balanced simultaneously. The goal is to select optimal solutions with the best possible trade-offs. Furthermore, we will enhance the metrics for performance (discrimination and calibration), fairness (Equalised Odd Difference, Demographic Parity, Equal Opportunity etc). We will include evaluation metrics (VSI, stability index, SHAP consistency Gap etc) to quantify the stability and consistency of overall explainability and reasoning particularly for each sub-group in sensitive variables. Implementation of systematic comprehensive hyper-parameter tuning with advanced EL, DL and transformer architectures to improve reliability on larger, complex and multi-domain datasets. Bias-aware experimentation will be introduced to ensure fairer decisions and more consistent explanations, particularly for sensitive demographic groups. Further development of hybrid models that combine the efficiency of EL with the interpretability of DL will be explored. In addition, we will investigate advanced SDG to enhance diversity and realism to address the imbalance problem for training of predictive models [31–34]. We fully recognise the associated risks of mode collapse, over-smoothing, and data hallucination. To mitigate these, we will incorporate distributional regularisation, adversarial training stabilisers, and fidelity–diversity trade-off metrics (e.g., Fréchet Distance, Coverage, and Density Scores) to ensure statistical authenticity of generated samples. Furthermore, all synthetic data will undergo feature correlation, causal, and fairness audits before integration into model training to avoid bias propagation. These innovations aim to enhance robustness while integrating fundamental components of responsible-AI that implements the theoretic principles of ethical-AI in different industries.

### Nomenclature

Following are abbreviations which we used in this manuscript:

**Table:** 

AdaBoost	Adaptive Boosting.
Adasyn Adaptive Synthetic (variant of SMOTE).	
B2S	Borderline2SMOTE.
Brier score	Calibration estimate
CPE	Comparative Performance Evaluation.
DT	Decision Tree.
1D-CNN One Dimension-Convolutional Neural Network.	
EDA	Exploratory Data Analysis.
ENN	Edited Nearest Neighbour.
F1 Score	Performance metric/ Harmonic Mean.
GAN	Generative Adversarial Network
GBDT Gradient Boosting Decision Tree	
LR	Logistic Regression.
Ln. R	Linear Regression
LightGBM	Light Gradient Boosting Model
Log_Loss	Entropy Loss (Calibration estimate)
LLM	Large Language Model
ML	Machine Learning
MLP Multilayer Perceptrons	
NN	Neural Network
PCA	Principal Component Analysis
RF	Random Forest.
ROS	Random Over-Sampling.
ROC-AUC	Receiver Operating Characteristic- Area Under Curve.
RUS	Random Under-Sampling.
SHAP	SHapley Additive ExPlanations
SLR	Systematic Literature Review.
SMOTE	Synthetic Minority Oversampling Technique.
SMOTEENN	Variant of SMOTE with edited nearest neighbours
SVM	Support Vector Machine.
TabNet Transformer based Neural Network	
XGBoost	Extreme Gradient Boosting.
XAI	Explainable Artificial Intelligence
